# Tularemia among Free-Ranging Mice without Infection of Exposed Humans, Switzerland, 2012

**DOI:** 10.3201/eid2101.140906

**Published:** 2015-01

**Authors:** Francesco C. Origgi, Barbara König, Anna K. Lindholm, Désirée Mayor, Paola Pilo

**Affiliations:** University of Bern, Bern, Switzerland (F.C. Origgi, D. Mayor, P. Pilo); and University of Zurich, Zurich, Switzerland (B. König, A.K. Lindholm)

**Keywords:** tularemia, zoonoses, house mouse, outbreak, human exposure, epizootic, Francisella tularensis, Switzerland, bacteria

## Abstract

The animals primarily infected by *Francisella tularensis* are rapidly consumed by scavengers, hindering ecologic investigation of the bacterium. We describe a 2012 natural tularemia epizootic among house mice in Switzerland and the assessment of infection of exposed humans. The humans were not infected, but the epizootic coincided with increased reports of human cases in the area.

Although the house mouse (*Mus musculus domesticus*) is a common model for infection with *Francisella tularensis* (*1*), no recent and detailed data are available about natural tularemia outbreaks in this species. Tularemia mainly affects rodents and lagomorphs (*2*), but because these species are rapidly consumed by scavengers (*3*), it is challenging to conduct investigations of the biologic cycle of *F. tularensis* in the environment. Furthermore, the disease mostly occurs sporadically, although outbreaks have been reported in animals and humans (*2*). We describe a natural outbreak of tularemia among a population of free-ranging house mice; the epizootic occurred in Switzerland in 2012 and was associated with possible human exposure. The mouse study was approved by the Swiss Animal Experimentation Commission (Kantonales Veterinäramt Zürich; permit 51/2010). 

## The Study

At the edge of a forest in the Canton of Zurich, Switzerland, a population of house mice is housed in a 72-m^2^ barn equipped with 40 nesting boxes. The population has been studied since 2002 to analyze the social structure and the population genetics of free-living house mice (*4*). The mice are monitored for research purposes every 2–3 days (*4*). Food, water, rodent bedding, and straw are provided ad libitum; mice are free to enter and exit the barn at any time. Larger animals are excluded from the barn, but other small mammals occasionally have been observed. In early June 2012, the mouse population in the barn was ≈360. 

Starting in early June 2012, increased numbers of mice were found dead in the barn. During May 2012–June 2013, a total of 69 carcasses were collected and stored frozen until necropsy was performed, beginning in mid-July 2012, after the initial peak of the outbreak (Figure). Full pathologic analysis could be performed on samples from 35/69 mice, of which 15 were PCR-positive for *F. tularensis*. The primary organs were collected and processed for histologic analysis. Pathologic investigation showed the presence of macroscopic and histologic changes. Skin lesions consistent with bite and fight wounds were observed in 7 mice, 1 of which was PCR-positive for *F. tularensis*; only gram-positive cocci were detected in the associated skin lesions of this mouse by light microscopy. Splenomegaly was observed in 23 mice. In 12 of these mice, splenomegaly was secondary to tularemia, and in 8, it was associated with amyloidosis and was frequently multisystemic. In 3 mice, splenomegaly was associated with amyloidosis and *F. tularensis* infection. Red to dark red mottling of the lung was observed in several affected mice, but obvious lung hemorrhages were observed in only 2 mice. The main histologic finding was the presence of multiple foci of necrosis in spleen, liver, and lung. In addition, frequent prominent thrombi and emboli were seen in lungs in association with severe vascular inflammatory infiltration and necrosis. 

**Figure:**
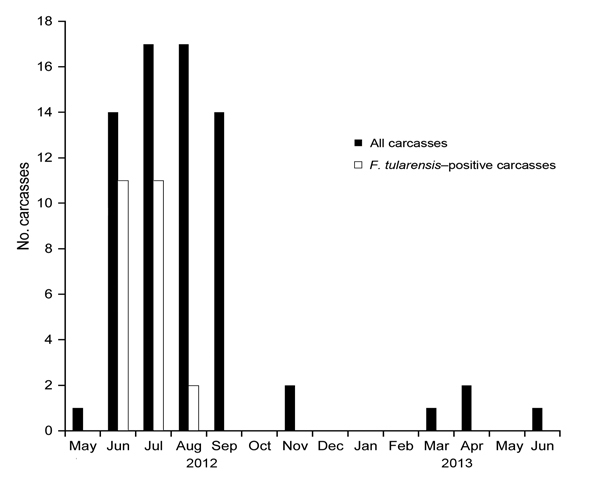
Monthly distribution of the number of carcasses of free-ranging house mice collected from a barn and the number positive for *F. tularensis*, Switzerland, May 2012–June 2013.

Overall, lung lesions consistent with *F. tularensis* infection (necrotizing pneumonia) were seen in 67% (10/15) of the *F. tularensis*–positive mice that were examined histologically; the lesions were observed throughout the outbreak and showed various degrees of size, extension, and severity. No similar lesions were observed in any of the *F. tularensis*–negative mice. A total of 69 samples were tested for *F. tularensis* by culture and direct PCR: 49 were spleen samples, including samples from mice not selected for pathologic investigation because of severe autolysis, and 20 were abdominal swab samples, which were used when the extreme grade of autolysis prevented the unambiguous identification or collection of the spleen following dissection (*5*). Spleen and swab samples from 24/69 mice were positive for *F. tularensis* by PCR. Tularemia cases were observed during June–August 2012 (Figure). Eight isolates from the spleens of 8 mice were identified as *F. tularensis* subsp. *holarctica* belonging to the lineage B.FTNF002–00; these isolates shared a single multilocus variable number tandem repeat analysis profile (*6*,*7*).

During the epizootic, 11 researchers regularly entered the barn during June 1–August 31, 2012, and were considered to have been exposed to *F. tularensis* (Table). On May 30 and June 29, 2012, influenza-like symptoms developed in researchers 1 and 3, respectively. *F. tularensis* antibodies persist in the blood, and serology is a standard method for diagnosing tularemia in humans. Thus, in late November or mid-December 2012 (≈6 months after the epizootic began), we obtained blood samples from the 11 researchers for serologic testing (VIRapid tularemia test; Vircell, Granada, Spain) (*8*): 10 samples were negative. The sample from researcher 3 had a positive test reaction and was further tested by microagglutination (*8*); dilutions of 1:40–1:640 were tested, and results were negative at 1:40. A second blood sample was obtained from researcher 3 in mid-March 2013 and was still positive by the rapid test but again showed no agglutination. Serologic cross-reaction with *Brucella* spp. was assessed and excluded. We then used the whole antigen from an outbreak isolate to perform IgM and IgG Western blots on the first and second serum samples from researcher 3: results were negative. 

**Table:** Estimated time 11 researchers spent in a barn inhabited by *Francisella tularensis*–infected house mice, Switzerland, June 1–August 31, 2012

Researcher no.	Total time in barn	
	Hours	Days
1	78	22
2	49	15
3	32	4
4	29	4
5	21	4
6	18	2
7	9	1
8	9	1
9	9	1
10	9	1
11	9	1

## Conclusions

Data concerning natural outbreaks of tularemia are difficult to obtain, especially from house mice, whose carcasses rarely remain available for collection because of predators and scavengers (*3*). In this study, a large population of mice could be monitored under natural conditions, in the absence of antimicrobial drug treatment, during a tularemia outbreak. PCR confirmed that during the ≈3-month outbreak of tularemia, 7% of the mouse population died from the disease. This number is relatively low considering the described high sensitivity of this species to *F. tularensis* (*1*); however, the number of exposed mice is unknown, and not all dead mice were available for testing. The lesions observed were similar overall to those previously reported (*9*). However, in our investigation, lung lesions were occasionally as severe or more severe than those observed in other tissues. The lung lesions varied in size, severity, and extension but remained consistent overall, suggesting a possible single route of infection and/or systemic spread.

Cannibalism (*10*) might have favored the transmission of bacteria within the mouse population, but most of the carcasses with skin wounds tested negative for *F. tularensis*. Thus, transmission through cannibalism is not likely. Transmission through arthropods may be possible because the study population naturally harbors fleas and mites; ticks have not been observed. 

Notification of *F. tularensis* outbreaks among rodents is essential, given the frequent presence of these animals in households and the consequent zoonotic potential of the pathogen (*11*). A unique aspect of this investigation is that we were able to evaluate humans with known exposure to infected animals. Eleven researchers entered the barn inhabited by house mice and monitored/handled the animals every 2–3 days without the use of specific personal protective equipment, except for disposable gloves; some of the mice were later found to be infected with *F. tularensis* (for more details about the monitoring/handling of animals, see [*4*]). The barn is a closed environment filled with bedding; mouse excrement is present on all surfaces and has the potential for aerosolization. Nevertheless, seroconversion was not detected in any of the researchers, bringing to question whether shedding of *F. tularensis* in urine and feces of mice is a key source of *F. tularensis* transmission for humans. 

Thus far, reports about *F. tularensis* shedding in rodents have had inconsistent findings (*12*–*14*). However, this is a crucial point to investigate because *F. tularensis* shedding through urine and feces would not only affect outdoor environments but also household environments via rodent infestation. Moreover, in Switzerland 150% more human tularemia cases were reported in 2012 than in 2011; the increase was mostly due to cases in the same area where the barn in this study is located (*15*), confirming the importance of monitoring sentinel animals for tularemia to better understand the ecology of *F. tularensis*.
